# Tomato Yellow Leaf Curl Virus V2 Protein Plays a Critical Role in the Nuclear Export of V1 Protein and Viral Systemic Infection

**DOI:** 10.3389/fmicb.2020.01243

**Published:** 2020-06-10

**Authors:** Wenhao Zhao, Shuhua Wu, Elizabeth Barton, Yongjian Fan, Yinghua Ji, Xiaofeng Wang, Yijun Zhou

**Affiliations:** ^1^Institute of Plant Protection, Jiangsu Academy of Agricultural Sciences, Key Lab of Food Quality and Safety of Jiangsu Province-State Key Laboratory Breeding Base, Nanjing, China; ^2^School of Plant and Environmental Sciences, Virginia Tech, Blacksburg, VA, United States

**Keywords:** *tomato yellow leaf curl virus* (TYLCV), V2 protein, V1 protein, nuclear export, viral systemic infection

## Abstract

Geminiviruses are an important group of circular, single-stranded DNA viruses that cause devastating diseases in crops. Geminiviruses replicate their genomic DNA in the nucleus and the newly synthesized viral DNA is subsequently transported to the cytoplasm for further cell-to-cell and long-distance movement to establish systemic infection. Thus, nucleocytoplasmic transportation is crucial for successful infection by geminiviruses. For *Tomato yellow leaf curl virus* (TYLCV), the V1 protein is known to bind and shuttle viral genomic DNA, however, the role of the V2 protein in this process is still unclear. Here, we report that the V1 protein is primarily localized in the nucleus when expressed but the nucleus-localized V1 protein dramatically decreases when co-expressed with V2 protein. Moreover, the V2-facilitated nuclear export of V1 protein depends on host exportin-α and a specific V1-V2 interaction. Chemical inhibition of exportin-α or a substitution at cysteine 85 of the V2 protein, which abolishes the V1-V2 interaction, blocks redistribution of the V1 protein to the perinuclear region and the cytoplasm. When the V2^C85S^ mutation is incorporated into a TYLCV infectious clone, the TYLCV-C85S causes delayed onset of very mild symptoms compared to wild-type TYLCV, suggesting that the V1-V2 interaction and, thus, the V2-mediated nuclear export of the V1 protein is crucial for viral spread and systemic infection. Our data point to a critical role of the V2 protein in promoting the nuclear export of the V1 protein and viral systemic infection, likely by promoting V1 protein-mediated nucleocytoplasmic transportation of TYLCV genomic DNA.

## Introduction

Geminiviruses are a group of plant viruses with a circular, single-stranded DNA genome. Viruses in this family cause devastating diseases in crop plants, leading to worldwide agricultural losses (Nakhla and Maxwell, 1997; Moriones and Navas-Castillo, 2000; Gafni, 2003; Fauquet et al., 2008; Glick et al., 2009; Fondong, 2019; Zeng et al., 2020). While viral protein synthesis occurs in the cytoplasm, replication of geminiviruses occurs in the nucleus of infected host cells (Hanley-Bowdoin et al., 2013). It is crucial that viral proteins involved in this replication enter the nucleus to execute their functions. In addition, newly synthesized viral genomic DNA is exported from the nucleus to the cytoplasm for further spread to adjacent cells followed by systemic infection through long-distance movement. Therefore, the nucleocytoplasmic shuttling of geminivirus proteins and genomic DNA is of great significance for viral systemic infection and a better understanding of the process will potentially provide new strategies to control viral infections.

Geminiviruses can be divided into two major groups based on their genomic components: one group is the monopartite geminiviruses, while the other group is the bipartite geminiviruses (Hanley-Bowdoin et al., 2013). The movement of bipartite geminiviruses requires two proteins, BV1 and BC1, which are encoded by DNA-B (Brough et al., 1988; Etessami et al., 1988; Padidam et al., 1995; Jeffrey et al., 1996; Sudarshana et al., 1998). BV1 is a nuclear shuttle protein and plays an important role in the nucleocytoplasmic shuttling of viral genomic DNA; BC1 facilitates cell-to-cell movement (Brough et al., 1988; Etessami et al., 1988; Jeffrey et al., 1996; Sudarshana et al., 1998; Lazarowitz and Beachy, 1999).

The genome of monopartite geminiviruses contains only one component, DNA-A. The possible mechanism for viral genomic DNA shuttling between the nucleus and the cytoplasm is not clear even though several monopartite geminiviruses have been examined, such as *Maize streak virus* (MSV) and *Tomato yellow leaf curl virus* (TYLCV) (Liu et al., 2001; Rojas et al., 2001; Gafni and Epel, 2002; Gorovits et al., 2016). It has been reported that the V1 protein, which is the coat protein (CP) of TYLCV, binds to and shuttles viral genomic DNA between the nucleus and cytoplasm in addition to packaging them in viral particles at a later stage (Boulton et al., 1989, 1993; Lazarowitz and Beachy, 1999). It was later reported that host proteins are also required for this process. Nuclear import receptor karyopherin α1 (KAPα) helps TYLCV enter the nucleus (Kunik et al., 1999; Yaakov et al., 2011), HSP70 (heat shock protein) is important for the TYLCV CP shuttle from cytoplasm into nucleus (Gorovits et al., 2016; Gorovits and Czosnek, 2017), and exportin-α is required for the nuclear export of the C4 protein of *Tomato leaf curl Yunnan virus* (TLCYnV) (Mei et al., 2018). In addition, nuclear shuttling of monopartite geminiviruses also involves viral proteins other than V1 protein, such as C4 or V2 protein, suggesting that a protein complex may be involved (Rojas et al., 2001, 2005; Mei et al., 2018). However, it is unclear what viral proteins and how they work together to accomplish the transportation between the nucleus and cytoplasm.

*Tomato yellow leaf curl virus* is a typical monopartite begomovirus in the family *Geminiviridae*. The single-stranded (ss) DNA genome has six open reading frames (ORFs) and an intergenic region (IR). Two ORFs (V1 and V2) are located on the viral strand and the other four ORFs (C1, C2, C3 and C4) are located on the complementary strand (Navot et al., 1991). Among them, V1 protein facilitates virion assembly and viral trafficking (Gafni, 2003; Rojas et al., 2001; Díaz-Pendón et al., 2010; Scholthof et al., 2011). For the nucleocytoplasmic transportation of TYLCV, V1 protein is well-known as a nuclear shuttle protein and for its role in binding viral genomic DNA (Kunik et al., 1998, 1999; Palanichelvam et al., 1998; Rojas et al., 2001). However, several lines of evidence suggest that other viral proteins, such as V2, are also involved (Kunik et al., 1999; Rojas et al., 2001; Jeske, 2009; Fondong, 2013; Hanley-Bowdoin et al., 2013; Sahu et al., 2014). Rojas et al. (2001) found that the efficiency of nuclear export of viral DNA was enhanced 20–30% in the presence of V2 protein, suggesting a role for V2 protein in the V1 protein-mediated nuclear export of viral genomic DNA. However, the mechanism whereby V2 protein facilitates the V1-mediated viral genomic DNA trafficking out of the nucleus is unknown.

In this study, we demonstrate that V2 protein affects the subcellular localization of V1 protein by dramatically decreasing the nucleus-localized V1 protein in *Nicotiana benthamiana* cells, possibly through host exportin-α (XPO I), which often mediates the nuclear export of proteins. A specific interaction between V2 and V1 proteins has been identified by co-immunoprecipitation (Co-IP) and bimolecular fluorescence complementation (BiFC). Substitutions for cystine 85 in V2 protein inhibit the V1-V2 interaction, block the effect of V2 protein on the subcellular localization of V1 protein, and cause delayed and mild symptom in plants. Our results indicate that the V2 protein interacts with V1 protein, promotes the nuclear export of V1 protein, and plays an important role in viral systemic infection.

## Results

### V2 Protein Affects the Nuclear Localization of V1 Protein

**FIGURE 1:**
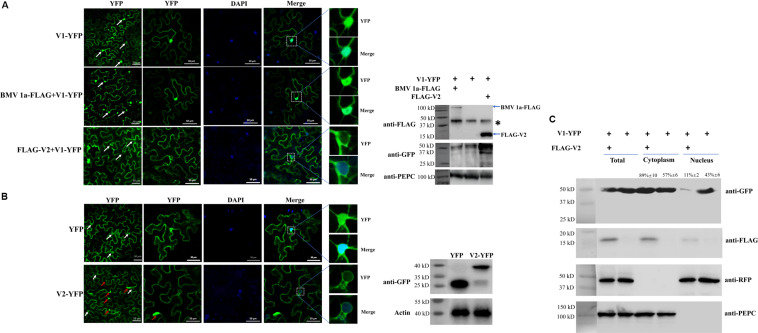
The effect of V2 protein on the nuclear distribution of V1 protein. **(A)** Localization of the V1 protein in the absence or presence of the V2 protein in *N. benthamiana* cells. V1-YFP expressed alone, co-expressed with BMV 1a-FLAG or FLAG-V2, detected either by confocal microscopy (left panel) or by western blotting using an anti-GFP polyclonal antibody (right panel). Arrows point to the nuclear areas in cells. DAPI stains DNA in the nucleus. PEPC serves as a control for equal loading of total lysates. Asterisk indicates a non-specific bands detected in all samples. Bars: 50 μm. **(B)** Localization of V2 in *N. benthamiana* cells. The expressed YFP or V2-YFP in epidermal cells of *N. benthamiana* leaves was detected either by confocal microscopy (left panel) or by western blotting using an anti-GFP polyclonal antibody (right panel). White arrows point to the nuclear areas in cells as shown in left column. Red arrows point to V2 aggregates. DAPI stains DNA in the nucleus. Bars: 50 μm. **(C)** Distribution of V1 protein in the absence or presence of FLAG-V2 in H2B-RFP transgenic *N. benthamiana* plants as analyzed by using a nuclear-cytoplasmic fractionation assay. Nuclei were purified using percoll density gradient centrifugation. Western blotting was conducted with antibodies specific to the indicated proteins. PEPC and H2B-RFP were used as a marker for the cytoplasmic and nuclear fraction, respectively. The intensity of protein signal was measured by using ImageQuant TL (GE healthcare), with levels of the cytoplasm plus the nucleus levels totaling 100%.

*Tomato yellow leaf curl virus* V1 protein is known as a nucleocytoplasmic shuttle protein that facilitates the transport of viral genomic DNA into and out of the nucleus. When expressed in cells of *N. benthamiana* by agroinfiltration as a YFP-tagged protein, V1-YFP, the signal was found in both the nucleus and cytoplasm at 40 h post agroinfiltration (hpai) (Figure 1A), consistent with its role in the nuclear transportation of viral genomic DNA. Among 100 cells with a clear nuclear region, strong YFP signal was detected in all cells (Figure 1A).

Since V2 protein was reported to facilitate the export of viral genomic DNA from the nucleus (Rojas et al., 2001), we tested whether V2 protein does so by promoting the nucleus export of the V1 protein. We first tested for the subcellular localization of V2 protein as a YFP-tag (V2-YFP) in *N. benthamiana* cells via agroinfiltration. The fluorescence signal was observed under a laser confocal microscope at 40 hpai. Large aggregates of V2-YFP were easily observed when high concentrations of agrobacteria were used for infiltration (Supplementary Figure S1). However, fewer aggregates were observed at OD_600_ = 0.5 for infiltration (Supplementary Figure S1). In addition, V2-YFP was mainly present in the cytoplasm and perinuclear regions, but a much weaker signal was also present in the nucleus (Figure 1B). To better examine the distribution of V2-YFP in the nuclear region, V2-YFP was expressed by infiltration of agrobacterium at OD_600_ = 0.5.

To further clarify the function of V2 protein in the nuclear export of TYLCV, we co-expressed FLAG-tagged V2 protein (FLAG-V2) with V1-YFP. Interestingly, a strong nuclear YFP signal was only detected in ∼13% of cells (*n* = 100). In about 87% of cells, only a weak fluorescence signal of the V1 protein was found in the nucleus compared to that of V1 protein alone (Figure 1A). To rule out the possibility that the weaker signal of V1-YFP in the nucleus was due to a decreased expression and/or stability in the presence of V2, we checked the accumulation of V1-YFP by western blotting. Our results showed that both V2 and V1 proteins were accumulated well when co-expressed (Figure 1A). Because V2 protein functions as a gene silencing suppressor, an increased accumulation of V1-YFP was noticed when it was co-expressed with FLAG-V2, indicating that the lower V1-YFP signal in the nucleus was not due to its decreased accumulation in the presence of FLAG-V2. To further rule out the possibility that overexpression of any protein may affect the distribution of V1 protein, we included replication protein 1a of *Brome mosaic virus* (BMV) (Diaz and Wang, 2014; Zhang et al., 2019). BMV 1a is an ER membrane-associated protein and redistributes specific host proteins to perinuclear ER membrane-invaginated viral replication complexes (Diaz and Wang, 2014; Diaz et al., 2015; Zhang et al., 2016). However, when co-expressed with V1-YFP, FLAG-tagged BMV 1a (BMV 1a-FLAG) did not show any effect on the localization of V1-YFP, as a strong signal was detected in the nucleus in all cells with detectable YFP signal (*n* = 100, Figure 1A), indicating that the redistribution of V1 protein is specifically mediated by V2 protein.

To confirm our visual observations, we performed a fractionation assay to separate the nucleus from the cytoplasm (Mei et al., 2018) and tested the localization of V1-YFP in the absence and presence of V2 protein. To this end, we expressed FLAG-V2 and V1-YFP in Histone 2B (H2B)-RFP transgenic plants (Martin et al., 2009). As shown in Figure 1C, we only detected H2B-RFP in the nuclear fraction but not the cytoplasmic fraction; a cytoplasmic marker, phosphoenolpyruvate carboxylase (PEPC), was only present in the cytoplasm fraction. Under such conditions, FLAG-V2 was primarily detected in the cytoplasm fraction but only weakly in the nucleus. Although V1-YFP was detected in both fractions when expressed alone, the amount in the nuclear fraction significantly decreased in the presence of FLAG-V2, which was consistent with the results based on fluorescence microscopy (Figure 1C). To provide a numeric reading, we set the sum of V1-YFP signal intensity in the cytoplasm and nucleus at 100%. In the absence of FLAG-V2, we found that 43% of V1-YFP was associated with the nuclear fraction but decreased to 11% in the presence of FLAG-V2. We concluded from these results that V2 protein is able to change the localization of V1 protein.

### V2 Protein Interacts With V1 Protein

**FIGURE 2:**
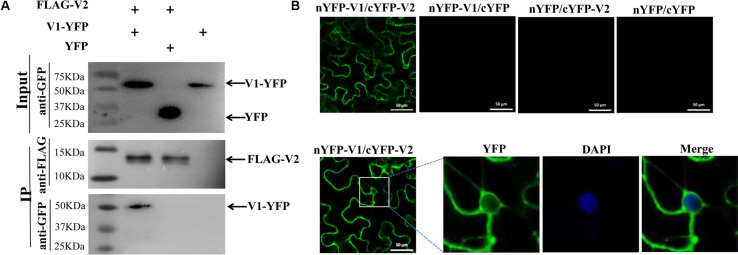
Identification of the interaction between V2 and V1 proteins. **(A)** Co-IP analysis of the interaction between FLAG-V2 and V1-YFP. *N. benthamiana* leaves were co-infiltrated with FLAG-V2 and V1-YFP (Lane 1), FLAG-V2 and YFP (Lane 2), or V1-YFP alone (Lane 3). Cell lysates were incubated with FLAG-trap beads and subsequently washed extensively. Samples before (Input) and after (IP) immunoprecipitation were analyzed using anti-GFP or -FLAG antibody. **(B)** BiFC assays for the interaction between V1 and V2 proteins in *N. benthamiana* cells. The V1-V2 interaction leads to a reconstituted fluorescence signal. DAPI stains DNA in the nucleus. Bars: 50 μm.

We then set out to understand the underlying mechanism by which V2 protein affects the subcellular localization of V1 protein by first testing whether there is an interaction between V2 and V1 proteins. We used a co-immunoprecipitation (Co-IP) assay because V1 protein is self-activating in the yeast two-hybrid (Y2H) system. FLAG-tagged V2 protein (FLAG-V2) was co-expressed with YFP or V1-YFP in *N. benthamiana*. Total protein extracts were subject to immunoprecipitation by using FLAG-trap beads, and the resulting precipitates were analyzed by an anti-GFP antibody or an anti-FLAG antibody. Although a similar amount of FLAG-V2 was pulled down with FLAG-trap beads, only V1-YFP, and not YFP, was pulled down along with FLAG-V2 (Figure 2A).

The fact that the V1 protein was co-precipitated with V2 protein suggests that V2 protein may bind to V1 protein to form a V1-V2 protein complex. To confirm the V1-V2 interaction and identify the location where V1 and V2 proteins may form a complex, we used a bimolecular fluorescence complementation (BiFC) assay. A positive interaction between nYFP-V1 and cYFP-V2 was observed in both the cytoplasm and perinuclear region, as indicated by the presence of reconstituted fluorescence (Figure 2B). We also noticed a faint fluorescence signal inside the nucleus. It should be noted that V1-YFP also localized in the cytoplasm and the perinuclear region when it was co-infiltrated with FLAG-V2 (Figure 1A), suggesting that V2 protein binds V1 protein at the perinucleus and the cytoplasm. No fluorescence signal was generated when nYFP-V1 and cYFP, or nYFP and cYFP-V2, or nYFP and cYFP were co-expressed (Figure 2B), reinforcing a specific interaction between V2 and V1 proteins in plant cells.

### V2 Protein Mediates the Nuclear Export of V1 Protein Through Host Exportin-α

**FIGURE 3:**
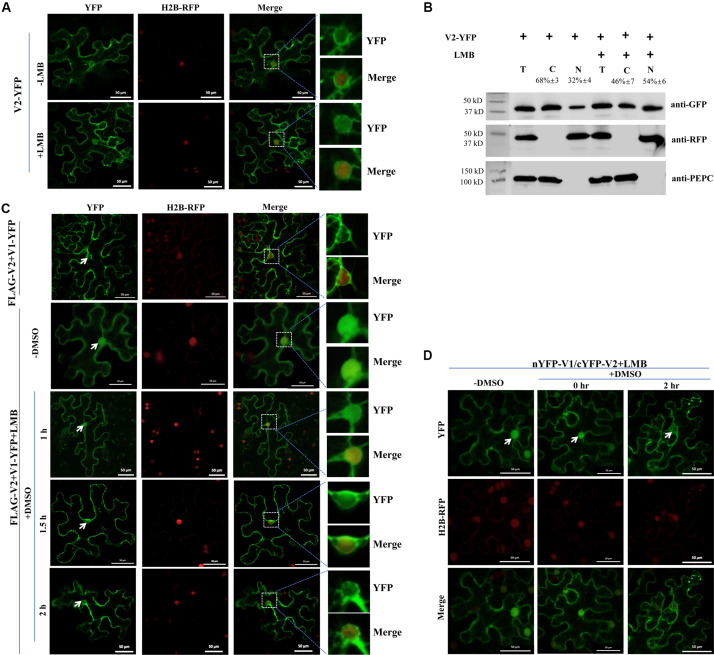
The V2-mediated nuclear export of V1 protein is dependent on exportin-α. **(A)** Subcellular distribution of V2-YFP without or with the LMB treatment in cells of the H2B-RFP transgenic *N. benthamiana* plant. Leaf tissues were first agroinfiltrated with V2-YFP for 40 h, followed by 10 nM LMB for 2 hours. The H2B-RFP signal represents the nucleus. Bars: 50 μm. **(B)** Nuclear-cytoplasmic fractionation analysis of the distribution of V2 with or without LMB treatment in H2B-RFP transgenic *N. benthamiana* plants. Western blotting analysis was conducted with antibodies specific to the indicated proteins. PEPC and H2B-RFP were used as a marker for the cytoplasmic and nuclear fraction, respectively. The intensity of protein signal was measured by using ImageQuant TL (GE healthcare), with levels of the cytoplasm plus the nucleus totaling 100%. **(C)** Subcellular distribution of V1-YFP co-expressed with FLAG-V2 upon the treatment of LMB and DMSO in H2B-RFP transgenic *N. benthamiana* cells. Leaf tissues expressing FLAG-V2 and V1-YFP were first infiltrated with 10 nM LMB for 2 hours and followed by infiltration of 0.5% DMSO to degrade LMB. The YFP signal was observed at specific time points as indicated. Arrows indicate the V1-YFP signal in or around the nucleus at different time points. H2B-RFP signal represents the nucleus. Bars: 50 μm. **(D)** Effects of LMB treatment on the V1-V2 interaction as shown by BiFC in H2B-RFP transgenic *N. benthamiana* plants. Plant tissues co-expressing nYFP-V1 with cYFP-V2 were treated with LMB for 2 h to inactivate exportin-α and then infiltrated with 0.5% DMSO to degrade LMB. Confocal micrographs were taken at the indicated time points. Arrows indicate the reconstituted YFP signal in or around the nucleus at different time points. The H2B-RFP signal represents the nucleus. Bars: 50 μm.

The fact that V2 protein hanged the nuclear localization of the V1 protein, raised the possibility that V2 protein might help V1 protein to export from the nucleus to the cytoplasm or block the entrance of V1 protein into the nucleus. Because the nuclear export of proteins is often mediated by exportin-α, we tested the subcellular localization of V2 protein upon treatment with leptomycin B (LMB), an inhibitor of exportin-α (Mathew and Ghildyal, 2017). As expected, the level of nucleus-localized V2-YFP increased after LMB treatment (Figure 3A), suggesting that V2 protein depends on exportin-α to move out of the nucleus. To confirm our observation, we performed a nuclear-cytoplasmic fractionation assay on H2B-RFP transgenic *N. benthamiana* plants expressing V2-YFP with or without LMB treatment. As shown in Figure 3B, H2B-RFP and PEPC were specifically detected, as expected, in the nuclear and cytoplasmic fractions, respectively. About 32% of the total V2-YFP accumulated in the nucleus but increased to 54% with the LMB treatment (Figure 3B), agreeing well with our imaging results (Figure 3A).

We also checked whether V2-mediated V1-YFP relocalization can be affected by the LMB treatment. Co-expressed with FLAG-V2, V1-YFP had very low accumulation in the nucleus (top panel, Figure 3C), but a strong nuclear signal was observed after treatment with LMB (second panel, -DMSO, Figure 3C), suggesting that V2-mediated nuclear relocalization of V1 protein is similar to the V2 protein export, which depends on exportin-α. The fact that both V1 and V2 proteins accumulated in the nucleus in the presence of LMB strongly suggested that V2 protein did not block the nuclear import of V1 protein but rather promoted the nuclear egress of V1 protein.

To confirm the specific effect of LMB on localizations of V1 and V2 proteins, we further infiltrated LMB-treated cells with 0.5% dimethyl sulfoxide (DMSO), which makes LMB unstable and thus, LMB becomes inactive (Mei et al., 2018). As expected, the V1-YFP signal was detected in the nucleus in the presence of FLAG-V2 and LMB at the beginning of the DMSO treatment (second panel, -DMSO, Figure 3C). However, the V1-YFP signal in the nucleus decreased gradually after a longer DMSO treatment that eliminated the inhibitory effect of LMB (Figure 3C).

To verify the nucleocytoplasmic shuttling of the V1-V2 complex, we performed a BiFC assay applying the same treatments as above. In the presence of LMB only, the reconstituted YFP signal was strongly detected in the nucleus (Figure 3D), indicating that the V1-V2 complex was also present in the nucleus as well as in the cytoplasm and perinuclear region (Figure 2B). After DMSO treatment for 2 h, the nucleus-localized YFP signal substantially decreased, which was accompanied by a ∼13% increase of the signal intensity in the cytoplasmic region, suggesting an exportin-α-mediated nuclear export of the V1-V2 complex (Figure 3D). These results indicated that the nucleocytoplasmic shuttling of V1 protein is dependent on the V2 protein and exportin-α.

### A V2 C85A Mutant Abolishes the V1-V2 Interaction

**FIGURE 4:**
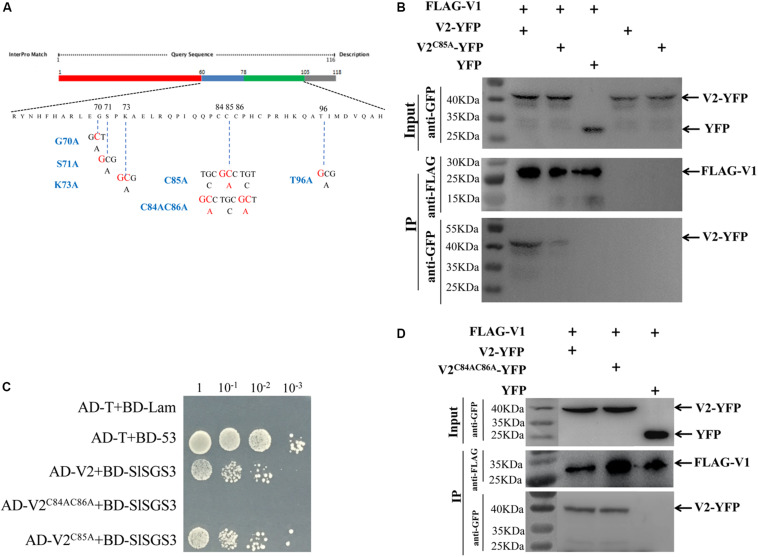
Identification of critical sites in the V2 protein responsible for the V1-V2 interaction. **(A)** Schematic illustration of the V2 protein. Nucleic acid and amino acid sequences of V2 mutants, V2^G70A^, V2^S71A^, V2^K73A^, V2^C85A^, V2^C84AC86A^, and V2^T96A^ are shown. **(B)** The interaction between V1 and wt V2 or V2^C85A^ is examined by a co-IP assay. The Co-IP assay was performed as in Figure 2A. **(C)** Y2H detecting possible interactions between SlSGS3 and V2^C85A^ or V2^C84AC86A^. V2^C85A^ and V2^C84AC86A^ were fused with a GAL4 activation domain (AD-V2^C85A^ and AD-V2^C84AC86A^), and SlSGS3 was fused to a GAL4-binding domain (BD-SlSGS3), respectively. AH109 cells co-transformed with the indicated plasmids were subjected to 10-fold serial dilutions and plated on synthetic-defined medium SD/-His/-Leu/-Trp medium to screen for positive interactions. Yeast cells co-transformed with AD-T + BD-53 or AD-T + BD-Lam serve as positive control or negative controls, respectively. **(D)** Co-IP assay to show the interaction between V1 and V2 or V2^C84AC86A^. The Co-IP assay was performed as in Figure 2A.

To verify that the V1-V2 interaction plays a crucial role in the nuclear export of V1 protein and to identify the approximate sites in V2 protein that are responsible for the interaction, we constructed six V2 mutants from a region (Figure 4A) that plays an important role in the V2-V2 self-interaction (Zhao et al., 2018) and the interaction between V2 and host proteins (Glick et al., 2008). We then tested their interactions with V1 protein using the Co-IP assay. Among the six V2 mutants, five of them (G70A, S71A, K73A, C84AC86A, and T96A) interacted with V1 protein as well as that of wt (data not shown). However, the V2^C85A^ mutant, which has a cysteine to alanine substitution in the residue at position 85, was not pulled down well along with FLAG-V1 because only a much weaker band was detected compare to that of wt V2-YFP (Figure 4B). The alanine substitution did not affect expression and stability of the V2^C85A^ mutant because V2-YFP and V2^C85A^-YFP accumulated at similar levels (top, Input panel, Figure 4B).

It is well-known that V2 protein is involved in PTGS by binding to tomato SGS3 (SlSGS3), an ortholog of the Arabidopsis SGS3 protein (Glick et al., 2008). It was confirmed that a double mutant of V2, V2^C84SC86S^, does not interact with SlSGS3 and lost its function as a suppressor of gene silencing (Glick et al., 2008). Given the fact that C85 is adjacent to C84 and C86, it is possible that V2^C85A^ may be dysfunctional not only in interacting with V1 protein but also with SlSGS3. To this end, we confirmed that V2^C85A^, but not V2^C84AC86A^, interacted with SlSGS3 in the Y2H system (Figure 4C), indicating that the C85A substitution specifically blocked the V1-V2 interaction but did not disrupt other functions of the V2 protein, such as the ability to interact with SlSGS3, which leads to a block of host gene silencing-mediated host defense. To directly test whether C85A may disrupt the activity of V2 protein in suppressing gene silencing, we transiently expressed wt V2 or V2^C85A^ in GFP-silenced 16c *N. benthamiana* plants (Nawaz-ul-Rehman et al., 2010). To induce gene silencing of GFP, *Agrobacterium* harboring a GFP expression vector was transiently infiltrated into the GFP transgenic line 16c at a 4-leaf stage. After five days post agroinfiltration, GFP expression was systematically silenced and no fluorescence signal was detected (Supplementary Figure S2a). These GFP-silenced leaves were then infiltrated with *Agrobacterium* harboring the vector expressing wt V2 or V2^C85A^. Strong GFP signal was recovered when the p19 protein of *Tomato bushy stunt virus* (TBSV) was expressed as a positive control (Supplementary Figure S2b). As expected, the expression of wt V2 led to a detectable GFP signal in the infiltrated region, even though not as strong as that of the p19-infiltrated area (Supplementary Figure S2b; Zrachya et al., 2007). Expressing V2^C85A^ also recovered GFP signal to the level similar to that of wt V2. Quantitative reverse transcription-PCR (qRT-PCR) further confirmed that similar levels of GFP transcripts were accumulated in the plants expressing wt V2 or the V2^C85A^ mutant (Supplementary Figure S2c), consistent with the note that C85A mutant had no effect on the gene silencing suppression activity of V2 protein.

To further confirm that C85, but not C84 and C86, is crucial for the V1-V2 interaction, we also tested the ability of V2^C84AC86A^ (Figure 4A) to interact with the V1 protein. The Co-IP assay indicated that the V2^C84AC86A^ mutant interacted with the V1 protein (Figure 4D). Taken together, the activities of the V2 protein in interacting with the V1 protein and SlSGS3 can be separated, where the C85A mutation blocks V2 protein’s interaction with V1 protein but not with SlSGS3.

### The V2^C85A^ Mutant Fails to Redistribute the V1 Protein

**FIGURE 5:**
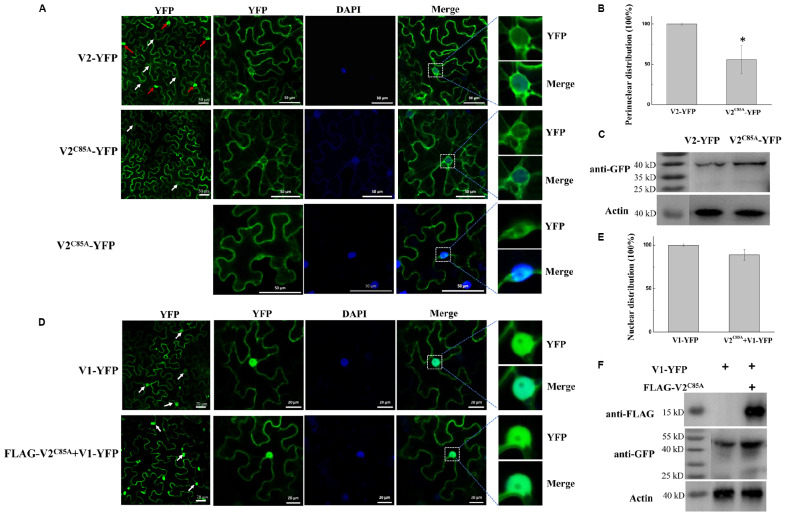
Characterization of the V2^C85A^ mutant. **(A)** Subcellular localization of V2 and V2^C85A^. DAPI stains DNA in the nucleus. White arrows in the left column point to the nuclear areas in cells. Red arrows point to V2 aggregates. Bars: 50 μm. **(B)** Quantification of perinuclear distribution of V2 and V2^C85A^. The number of cells with perinuclear distribution in different samples as in a. Experiments were repeated three times and 30 cells were observed in each repeat. Values represent percentages of cells with a perinuclear distribution of YFP signal ± SD (standard deviation). Data were analyzed using Student’s *t*-test and asterisks denote significant differences between V2-YFP- and V2^C85A^-YFP-infiltrated leaves (*P < 0.05). **(C)** Western blot analysis showing accumulated V2 and V2^C85A^ using anti-GFP polyclonal antibody. Actin serves as a control for equal loading. **(D)** Localization of V1-YFP expressed alone or co-expressed with V2^C85A^ in *N. benthamiana* leaves. Arrows in the left column point to the nuclear areas in cells. Bars: 20 μm. **(E)** Comparison of the nucleus-localized V1-YFP in the absence or presence of V2^C85A^. At least 150 cells were analyzed from three independent repeats. Values represent the mean ± SD in plants infiltrated with V1-YFP in the absence or presence of V2^C85A^. The data were analyzed using Student’s *t*-test. **(F)** The accumulated V1-YFP and FLAG- V2^C85A^ as shown by western blot analysis.

After confirming that V2^C85A^ accumulated well and interacted with SlSGS3 but not V1 protein, we next checked the localization of V2^C85A^ by expressing YFP-tagged V2^C85A^ (V2^C85A^-YFP) in *N. benthamiana* cells. In 56% of cells expressing V2^C85A^-YFP (Figure 5B), a fluorescence signal was observed in the cytoplasm and perinuclear region (Figure 5A), similar to that of wild-type (wt) V2-YFP. In 44% of cells, however, the fluorescence signal was more spread than that of V2-YFP and was also observed in an elongated region beyond the DAPI-stained nucleus (Figure 5A). The nature of the localization remains to be determined. It needs to note that the C85A mutation did not affect the expression or stability of V2-YFP because V2^C85A^-YFP accumulated at a similar level as V2-YFP (Figures 4B, 5C). These results indicated that C85 has some effects on the perinuclear localization of the V2 protein.

To test the effect of C85A substitution in V2 protein on the localization of V1, FLAG- V2^C85A^ was co-expressed with V1-YFP in *N. benthamiana* cells. A strong V1-YFP signal was detected in the nucleus in the presence of FLAG- V2^C85A^, similar to that when V1-YFP was expressed alone (Figure 5D). Among 50 cells that were examined for the localization of V1 protein, no obvious difference in the V1-YFP distribution pattern was observed in the absence or presence of V2^C85A^ (Figure 5E), suggesting that V2^C85A^ was not able to affect the nuclear localization of V1 protein. Because V1-YFP accumulated at a higher level in the presence of V2^C85A^ compared to that in the absence of V2^C85A^ (Figure 5F), we propose that the disrupted V1-V2 interaction is responsible for the failed redistribution of V1-YFP. However, we cannot totally rule out the possibility that other uncharacterized functions might be affected by alanine replacement.

### A C85 Substitution in V2 Protein Delays Viral Systemic Infection

To assess the role of the V1-V2 interaction in viral infection, we incorporated a substitution in the C85 of the V2 ORF in the backbone of an infectious TYLCV clone. As the V2 ORF overlaps with the V1 ORF in the TYLCV genome, mutations in V2 may affect V1 amino acid sequence. To ensure that a specific change in C85 has no effect on the V1 protein in TYLCV genome, the C85S mutation, instead of the C85A mutation, was introduced into a TYLCV clone to generate TYLCV-C85S. It needs to be addressed that the V2^C85S^ mutant did not interact with V1 protein (Supplementary Figure S3a), but interacted with SlSGS3 (Supplementary Figure S3b), and in turn, maintained its activity as a suppressor of gene silencing (Supplementary Figures S2b,c). Most importantly, the V2^C85S^ mutant did not affect the subcellular localization of V1-YFP (Supplementary Figure S3c), similar to the properties of V2^C85A^. TYLCV-C85S and TYLCV were subsequently used to inoculate tomato (*Solanum lycopersicum*) and *N. benthamiana* plants.

**FIGURE 6:**
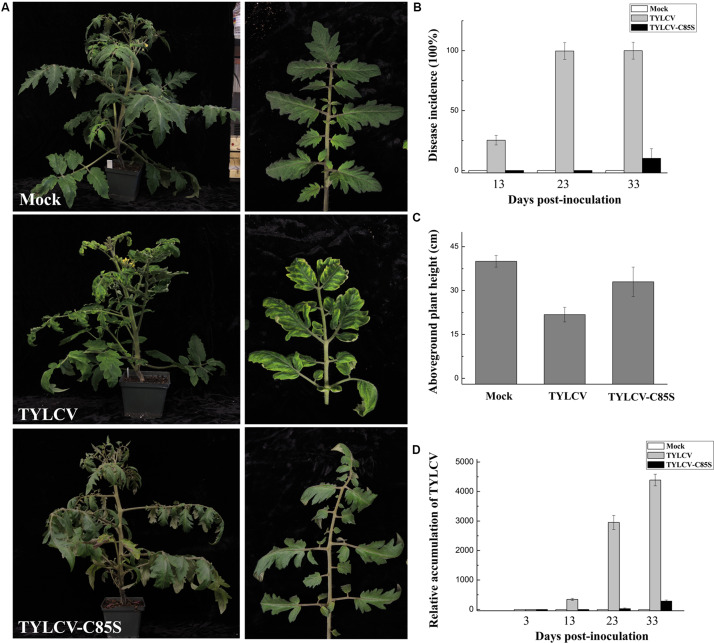
Effects of the C85S mutation on viral infection and viral accumulation in TYLCV-inoculated tomato plants. **(A)** Symptoms in plants that were agroinoculated with wt TYLCV or TYLCV-C85S at 33 dpi. Mock represents mock-inoculated plants. Enlarged images show the yellowing and curling leaves. **(B)** The time course of wt TYLCV or TYLCV-C85S infection. Values representing percentages of systemically infected plants at different dpi are given as mean ± SD of triplicate experiments. In each experiment, 15 plants were inoculated and four independent repeats were performed. **(C)** The aboveground plant heights of Mock-, TYLCV-, or TYLCV-C85S-inoculated tomato plants as measured at 33 dpi. **(D)** The accumulated viral DNA in plants as measured by qPCR. Accumulated levels of viral DNA were tested in TYLCV-, TYLCV- C85S-, or mock-inoculated plants at 3, 13, 23, and 33 dpi. Total DNA was extracted from newly emerged systemic leaves. Values represent the mean relative to the Mock-treated plants (*n* = 3 biological replicates) and were normalized with *SlActin* as an internal reference.

Fifteen tomato plants were inoculated with either wt TYLCV or TYLCV-C85S. Symptoms such as chlorosis on leaves were obviously observed at 13 days post-infection (dpi) on tomato plants inoculated with wt TYLCV but not in the plants inoculated with TYLCV-C85S. The vast majority of TYLCV-C85S-inoculated tomato plants remained symptomless even at 33 dpi and only 1-2 plants among 15 eventually developed mild symptoms, such as leaf yellowing (Figures 6A,B). In addition, the average height of TYLCV-C85S-inoculated tomato plants was 32 ± 3.4 cm, which was higher than that of TYLCV-inoculated plants at 22 ± 2.0 cm (Figure 6C). Real-time PCR showed that levels of viral genomic DNA were much lower in systemic leaves of plants inoculated with TYLCV-C85S than those in plants inoculated with wt TYLCV at 13, 23, and 33 dpi (Figure 6D). These were consistent with the presence of typical symptoms in wt TYLCV-infected plants but no or very mild symptoms in TYLCV-C85S-infected plants at 33 dpi (Figures 6A,B).

**FIGURE 7:**
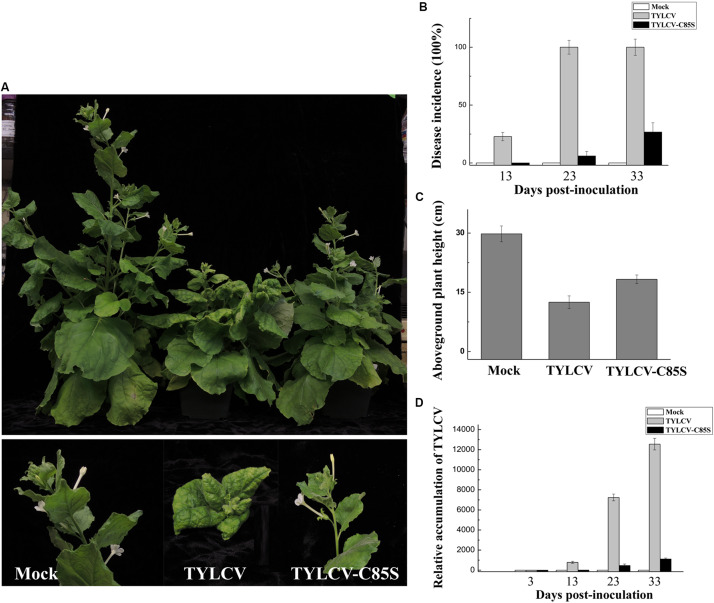
Effects of the C85S mutation on viral infection and viral accumulation in TYLCV-inoculated *N. benthamiana*. **(A)** Symptoms in plants that were agroinoculated with wt TYLCV or TYLCV-C85S at 33 dpi. Mock represents mock-inoculated plants. **(B)** The time course of wt TYLCV or TYLCV-C85S infection. Values represent percentages of systemically infected plants as performed in Figure 6B. **(C)** The aboveground plant heights of mock-, TYLCV-, or TYLCV-C85S-inoculated *N. benthamiana* plants as measured at 33 dpi. **(D)** The accumulated viral DNA in plants was measured by qPCR. qPCR was done as Figure 6D. *NbActin* as an internal reference.

Similar results were also obtained in TYLCV-C85S-inoculated *N. benthamiana* plants. All wt TYLCV-inoculated plants showed symptoms as early as 13 dpi, such as leaf yellowing and curling as shown in Figure 7A. However, only 3-4 out of fifteen plants inoculated with TYLCV-C85S showed mild symptoms (Figure 7B). We did notice that plants infected by TYLCV-C85S were shorter (18 ± 1.1 cm) compared to healthy plants at 30 ± 2.0 cm (Figure 7C). The accumulated TYLCV genomic DNA (Figure 7D) in systemic leaves of TYLCV-C85S-inoculated plants were much lower than those in wt TYLCV-inoculated plants at 13, 23, and 33 dpi, consistent with the presence of typical symptoms in systemic leaves of wt TYLCV-inoculated plants but no or very mild symptoms in systemic leaves of TYLCV-C85S-inoculated plants (Figure 7A).

Collectively, these results showed that the mutation at C85 of the V2 protein causes significantly low levels of virus accumulation in the systemic leaves and a dramatic decrease in the infection efficiency with delayed and mild symptoms.

## Discussion

Because genome replication of geminiviruses takes place in the nucleus of the infected host cells (Hanley-Bowdoin et al., 2013), it is crucial to transport the viral offspring DNA from the nucleus back to the cytoplasm for intracellular, cell-to-cell, and long-distance movement. In bipartite geminiviruses, it is well-known that the BV1 protein facilitates trafficking of the viral genome into and out of the host nucleus (Brough et al., 1988; Etessami et al., 1988; Sudarshana et al., 1998; Jeffrey et al., 1996; Ye et al., 2015). In monopartite geminiviruses, it has been reported that the V1 protein mediates the import and export of viral DNA (Kunik et al., 1998, 1999; Rojas et al., 2001). However, V1 protein might not be the only viral protein that is involved in the nucleocytoplasmic shuttling of TYLCV. Previous reports based on triple microinjection experiments revealed that the nuclear export of viral DNA was enhanced 20–30% in the presence of V2 and V1 proteins compared to that with V1 protein only (Rojas et al., 2001). However, the mechanism by which the V2 protein promotes viral DNA export is unclear. Our results suggest that V2 protein may facilitate viral DNA export by interacting with V1 protein and promote the nuclear export of V1 protein.

In this study, we found that the V2 protein localized primarily in the perinuclear region and the cytoplasm (Figure 1B). A very weak signal was also present in the nucleus (Figures 1B,C), but upon treatment with the exportin-α inhibitor LMB, the amount of nucleus-localized V2 protein increased significantly (Figure 3A), suggesting that V2 protein shuttles between the nucleus and the cytoplasm but is quickly exported out of the nucleus via exportin-α. It is unclear, however, how V2 protein is imported into the nucleus.

Our work found that V1 protein was primarily localized in the nucleus when expressed alone, but the nucleus-localized V1 disappeared when co-expressed with V2 protein (Figure 1A), suggesting that V2 either promoted the nuclear export of V1 or inhibited the nuclear entry of V1 protein. Although we cannot totally rule out the possibility that V2 may prevent V1 protein from entering the nucleus, our data suggest that V2 protein plays a critical role in the nuclear export of V1 protein as the nucleus-localized V1 diminished when V2 was present (Figure 1A). In addition, V1 protein was still accumulated in the nucleus when expressed along with V2 in the presence of LMB (Figure 3C), suggesting that V2 protein enhances the nuclear export of V1 but not the nuclear import. We also showed that the specific V1-V2 interaction is closely correlated with V1 trafficking. The V1-V2 interaction primarily occurred in the perinuclear region and the cytoplasm (Figure 2B) but was strongly detected in the nucleus upon LMB treatment (Figure 3D), suggesting that they may be in a complex or complexes throughout viral replication and movement in infected cells. In addition, it also suggests that LMB only specifically blocked the V2’s transport out of the nucleus but had no effect on the V1-V2 interaction. However, our data were not able to determine whether V2 protein mediates the nuclear import of V1 protein. In addition, our results do not rule out the possibility that other viral proteins, such as the C4 protein, may also be involved in this process.

Cysteine85 of V2 protein was found to be crucial for the V1-V2 interaction because substitutions of Cys85 with alanine (Figure 4B) or serine (Supplementary Figure S3a) led to a substantially inhibited interaction with V1 and thus, its ability to facilitate V1’s transport out of the nucleus (Figures 5D,E for C85A and Supplementary Figure S3c for C85S). Because V1 protein is known for binding to and facilitating nucleocytoplasmic trafficking of viral DNA (Kunik et al., 1998, 1999; Rojas et al., 2001), and because V2 facilitates the nuclear export of viral DNA along with V1 protein (Rojas et al., 2001), we propose that the V2-promoted nuclear export of viral DNA is likely via the V1-V2 interaction. Our hypothesis is consistent with our results that the TYLCV-C85S mutant, which has the C85S mutation incorporated into an infectious TYLCV clone, led to the delayed onset of symptoms and reduced viral accumulation (Figures 6, 7). These results indicated that C85 of V2 protein plays an important role in viral systemic infection.

In monopartite geminiviruses, V2 protein is a multifunctional protein that is involved in suppressing host PTGS and TGS, pathogenicity and systemic infection (Wartig et al., 1997; Zrachya et al., 2007; Bar-Ziv et al., 2012; Wang et al., 2014, 2018). Substitution in cysteine 85 may affect functions other than its interaction with V1 protein, especially since Cys84 and Cys86 are critical for interacting with SlSGS3 and the suppression of gene silencing (Glick et al., 2008). We found that even though the C85A (Figure 4B) or C85S (Supplementary Figure S3a) mutants failed to interact with V1 protein and thus, V1’s trafficking out of the nucleus (Figures 5D,E; Supplementary Figure S3c), both maintained their activity as a suppressor of gene silencing (Figure 4C; Supplementary Figures S3b, S2). These results are also consistent with the notion that the C85S mutation delays viral systemic infection by affecting V1-mediated viral genomic DNA transportation from the nucleus to the cytoplasm, not by disturbing gene silencing-mediated host defense. However, we cannot totally rule out the possibility that other V2-mediated viral infection step(s) besides viral DNA trafficking are affected by the C85S mutation.

Our data also showed that the V2 C84AC86A double mutant interacted with V1 (Figure 4D) but not SlSGS3 (Figure 4C), indicating that C84 and C86 are not related to V2’s ability to interact with V1 protein. Our results therefore revealed that motifs responsible for V1’s nuclear export and the suppressor activity of gene silencing may be independent from one another.

The TYLCV V2 protein was found to be associated with large cytoplasmic aggregates (Moshe et al., 2015; Zhao et al., 2018). We demonstrated that the number of V2 aggregates is related to the *Agrobacterium* concentration used for infiltration (Supplementary Figure S1). It is well known that many viruses induce the formation of aggregates/inclusion bodies in the infected cells, which might be involved in viral replication and eventually related to viral infection (Wileman, 2006; Gorovits et al., 2013). However, the aggregates induced by the overexpressed V2 protein localized primarily in the cytoplasm but not the nucleus, where TYLCV replication occurs, suggesting that these aggregates might not be associated with viral replication. The relationship between V2 aggregates and viral infection needs further study.

Our results indicated that V2 protein binds to V1 protein and facilitates the nuclear export of V1 protein. During TYLCV infection, V1 mediates both nuclear import and export of viral DNA. The equilibrium between nuclear targeting and egress is changed upon completion of replication and the V1-V2 interaction can improve the nuclear export of the V1-DNA complex. Thus, viral DNA will be preferentially transported out of the nucleus for subsequent infection events. In the presence of the V2^C85S^ mutant, the nuclear export of V1 is slowed down or eliminated and therefore, viral DNA and subsequent viral cell-to-cell and systemic movement is delayed. However, we cannot totally rule out that the V1-V2 complex is also required for intracellular, cell-to-cell, and/or long-distance movement besides nuclear export of V1 protein and V1-mediated viral offspring DNAs.

**FIGURE 8:**
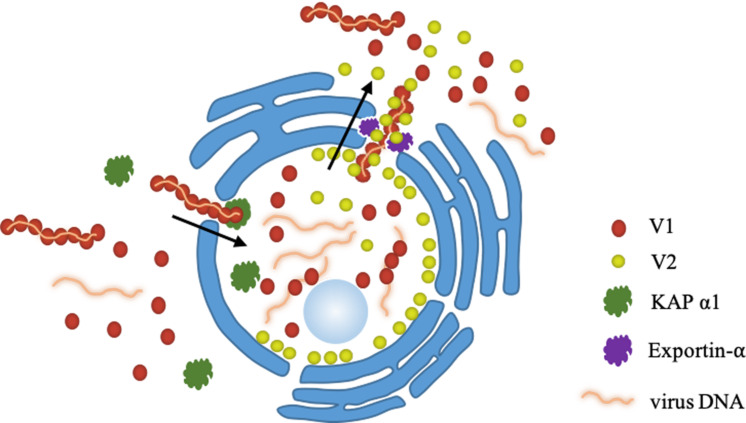
A working model proposed for the V2-mediated nucleocytoplasmic trafficking of the V1 protein. Viral genomic DNAs are bound by V1 and import into the nucleus with the help of KAP α1 via the specific interaction between V1 and V2, leading the formation of a V2-V1-ssDNA complex. With the help of exportin-α, V2 facilitates the V1-ssDNA complex to exit the nucleus to the perinucleus and the cytoplasm.

Based on our findings here, we propose a working model for the role of V2 protein in V1-mediated nuclear export of TYLCV genomic DNA (Figure 8). When offspring viral genomic DNA is produced in the nucleus, they are bound by V1 protein (Palanichelvam et al., 1998). A V2-V1-viral DNA complex is subsequently formed via a specific interaction between V1 and V2 and, with the help of exportin-α, V2 facilitates the V1-viral DNA complex to egress from the nucleus to the perinucleus and the cytoplasm with enhanced efficiency. Eventually, TYLCV spreads to adjacent cells and upper leaves, which results in a systemic infection. The infection efficiency and the accumulation of TYLCV in the systemic leaves are dramatically inhibited with a defective V2-V1 interaction.

In summary, our results reveal that one mechanism of V2 protein’s involvement in viral DNA transportation is to promote V1-mediated viral egress from the nucleus to the perinuclear region and the cytoplasm through a specific interaction with V1 protein, in the form of a V2-V1-viral DNA complex, and via host exportin-α. However, whether V2 promotes the ability of V1 to bind viral DNA and whether the V1-V2 interaction works after nuclear transportation require further research.

## Experimental Procedures

### Plasmid Construction

The coding sequences of TYLCV V2 and V1 genes were amplified from the cDNA of a TYLCV-infected tomato plant from Jiangsu Province, China (GenBank accession number GU111505) (Ji et al., 2008), using corresponding primers (Supplementary Table S1). Site-specific mutants of V2^G70A^, V2^S71A^, V2^K73A^, V2^C85A^, V2^C84AC86A^, V2^C85S^, and V2^T96A^ were synthesized (Invitrogen, China) and confirmed by sequencing (Figure 4A). For more details, see the electronic supporting information.

### Subcellular Localization of Proteins

The expression vectors p1300-YFP, V2-YFP, V1-YFP, V2^C84AC86A^-YFP, V2^C85A^-YFP and V2^C85S^-YFP were individually introduced into *A. tumefaciens* strain GV3101 through electroporation. Leaves of 4-week-old *N. benthamiana* were infiltrated with *A. tumefaciens* harboring the designated constructs. At 40 hpai, leaves were excised and YFP fluorescence was examined in epidermal cells using confocal microscopy (Zeiss LSM 710). The microscope was configured with a 458–515 nm dichroic mirror for dual excitation and a 488-nm beam splitter to help separate YFP fluorescence.

### Bimolecular Fluorescence Complementation (BiFC) Assay

Bimolecular Fluorescence Complementation experiments were performed as previously described (Shen et al., 2012) with minor modifications. The constructs nYFP-V1 and cYFP-V2 were introduced individually into GV3101 by electroporation. After overnight growth and activation, agrobacterium cultures were combined and infiltrated into leaves of *N. benthamiana*. After agroinfiltration, *N. benthamiana* were grown in a growth chamber with a 16 h light/8 h dark cycle. YFP fluorescence was observed and photographed using confocal microscopy (Zeiss LSM 710) at 48 hpai. YFP was observed under a mercury lamp light using a 488-nm excitation filter. Photographic images were prepared using ZEN 2011SP1.

### Co-immunoprecipitation Assay

The Co-IP assay was performed as previously described (Zhao et al., 2018). The infiltrated *N. benthamiana* leaves were harvested at 40 hpai. Proteins were extracted in IP buffer (40 mM Tris-HCl at pH 7.5, 100 mM NaCl, 5 mM MgCl_2_, 2 mM EDTA, 2 × EDTA-free proteinase inhibitor, 1 mM PMSF, 4 mM DTT, 1% glycerol, 0.5% Triton-X100). After centrifugation, the supernatant was mixed with FLAG-trap beads (Sigma, United States). After 1 h incubation at 4°C, the beads were washed with IP buffer and resuspended in 2 × SDS loading buffer. The samples were loaded onto a 12% (vol/vol) SDS/PAGE gel and target proteins were detected using a polyclonal anti-GFP antibody (GenScript, United States) or a monoclonal anti-FLAG (Sigma, Unites States) antibody.

### Yeast Two-Hybrid Assay

The yeast two-hybrid system was used to examine interactions between V2, V2^C85A^, V2^C84AC86A^ and SlSGS3. V2, V2^C85A^, and V2^C84AC86A^ were cloned into the activation domain (AD) vector and SlSGS3 was cloned into the vector harboring the DNA binding domain (BD). Both constructs were transformed into the yeast strain AH109. The plasmid pair of BD-53 and AD-recT served as a positive control, while BD-Lam and AD-recT was used as a negative control. Transformants were grown at 30°C for 72 h on synthetic defined medium lacking Histine, Leucine and Tryptophan (SD/-His/-Leu/-Trp) to test protein-protein interactions.

### Nuclear-Cytoplasmic Fractionation Assay

Nuclear-cytoplasmic fractionation assays were performed as described previously (Wang et al., 2011) with minor modifications. Infiltrated leaves were harvested and mixed with 2 mL/g of lysis buffer (20 mM Tris-HCl, pH 7.5, 20 mM KCl, 2 mM EDTA, 2.5 mM MgCl_2_, 25% glycerol, 250 mM Sucrose, 5 mM DTT, 10 mM protease inhibitor). The centrifuged pellet was resuspended with 500 μL of NRB2 and overlaid on top of 500 μL NRB3. The final nuclear pellet was resuspended in lysis buffer. As quality controls for the fractionation assays, PEPC protein and H2B-RFP were used as a cytoplasmic and a nuclear marker, respectively. For more details, see the electronic supporting information.

### Leptomycin B Treatment Assays

Leptomycin B (LMB) Treatment Assays were performed as previously described (Mei et al., 2018) with minor modifications. LMB (Fisher Scientific, United States) was dissolved in ethanol to prepare 10 mM stock solutions. For *in vivo* treatment of *N. benthamiana* leaves, stock solutions were diluted in water to prepare a working solution of 10 nM LMB. Agroinfiltrated *N. benthamiana* leaves expressing the protein of interest at 40 hpai were infiltrated with 10 nM LMB. Two hours after LMB treatment, the infiltrated leaves were cut and mounted on a glass slide for confocal imaging. When needed, DMSO was further infiltrated into LMB-treated leaves and tissues were harvested at the specified time points.

### TYLCV Constructs for *Agrobacterium*-Mediated Inoculation

To make a DNA clone of TYLCV containing V2^C85S^, a full-length TYLCV mutant, TYLCV-C85S was synthesized (Invitrogen, China). The TYLCV-C85S and wt TYLCV infectious clone were constructed as previously described (Zhao et al., 2018). For more details, see the electronic supporting information.

*Agrobacterium* cultures harboring TYLCV constructs were injected into the stem of tomato and *N. benthamiana* with a syringe. Inoculated plants were grown in an insect-free cabinet with supplementary lighting corresponding to a 16-hour light and 8-hour dark schedule.

### Quantitative PCR

Total DNA was extracted from mock (*Agrobacterium*-carrying empty vector)-, wt TYLCV- or TYLCV-C85S-infiltrated tomato or *N. benthamiana* leaves at different time points. RCR reaction mixes consisted of 6 μl of SYBR Green supermix (BIO-RAD, United States), 0.10 μl of each primer (10 pmol) and 1.5 μl of DNA sample (10 ng/μl) in a total volume of 12 μl.

PCR reactions were done in an Applied Biosystems 7500 (Life Technologies) real-time PCR detection system. *SlActin* or *NbActin* was used as an internal control for tomato or *N. benthamiana*, respectively. Data analysis was performed using Applied Biosystems 7500 software version 2.0.6.

## Data Availability Statement

The coding sequences of TYLCV V2 and V1 can be accessed at the NCBI database with the accession number GU111505.

## Author Contributions

YZ, YJ, XW, and WZ designed the project. WZ, SW, and EB conducted experiments. All authors analyzed the data and reviewed the manuscript. WZ, YJ, and XW wrote the manuscript.

## Conflict of Interest

The authors declare that the research was conducted in the absence of any commercial or financial relationships that could be construed as a potential conflict of interest.
