# Supplementary material for: Tomato Yellow Leaf Curl Virus V2 Protein Plays a Critical Role in the Nuclear Export of V1 Protein and Viral Systemic Infection
Source: Front Microbiol. 2020 Jun 10;11:1243. doi: 10.3389/fmicb.2020.01243 (PMC7297916; doi:10.3389/fmicb.2020.01243)
Supplement: Supplementary file 1 [file Data_Sheet_1.docx]

**Supporting Information**

This file contains

-Supplementary Methods

-Supplementary Table

-Supplementary Figure

**Supplementary Methods**

**Plant Materials and Growth Conditions**

All agro-infiltration experiments were performed in wild-type (wt) or a *N. benthamiana* transgenic line expressing the nuclear maker H2B, H2B-RFP (red fluorescent protein fused to the C terminus of histone 2B) (Martin et al., 2009). Plants were grown in a growth chamber at 26°C (16 h, light) and 22°C (8 h, dark) for 4-6 weeks before being infiltrated with the agrobacterium. After infiltration, the plants were kept under the same growth conditions and analyzed at the specified time points.

**Plasmid Construction**

To investigate the subcellular localization, the TYLCV V2 (*Bgl*II), V1 genes (*Bam*HI) and V2^C85A^, V2^C84AC86A^, V2^C85S^ were amplified using specific primers as listed in Supplementary Table S1. Yellow fluorescent protein (YFP) tag was inserted between the CaMV 35S promoter and the 35S terminator (35St) in the pCambia1300 binary vector to construct the p1300-YFP vector as previously described (Zhao et al., 2018). All amplified PCR products were individually inserted into the *Bam*HI site of the p1300-YFP vector to fuse in frame with YFP at the N-terminus to generate V2-YFP, V1-YFP and V2^C85A^-YFP, V2^C84AC86A^-YFP, V2^C85S^-YFP.

To make BiFC vectors, full-length coding sequences of V2 and V1 genes were amplified using the primers listed in Supplemental Table S1, V1 was cloned into the *Bam*HI site as a fusion with the N-terminal fragment of YFP and V2 was cloned into the *Bam*HI site as a fusion with the C-terminal fragment of YFP, resulting in nYFP-V1 and cYFP-V2.

FLAG tagged V2 and V1 were amplified by PCR using specific primers (S1 Table) and inserted into the *Bam*HI site between the 35S promoter and the 35St in the pCambia1300 binary vector to generate FLAG-V2 and FLAG-V1 for the Co-IP experiments. C-terminally FLAG-tagged BMV 1a was under an enhanced CaMV 35S promoter in the pCambia1300 binary vector. BMV 1a-FLAG supports full replication in plants.

For the yeast two-hybrid assay, V2, V2^C85A^, V2^C84AC86A^ and V2^C85S^ were amplified and inserted into the *Nde*I/*Eco*RI-digested pGADT7 vector. SlSGS3 was cloned into the *Nde*I/*Bam*HI-digested pGBDT7 vector.

**Agro-infiltration Assays in *N. benthamiana***

Target vectors were transformed into *A. tumefaciens* strain GV3101 by electroporation or heat shock. Agrobacterial cultures were harvested when the OD_600_ was approximately 0.8–1.0, collected by centrifugation, resuspended in the induction buffer (10 mM MgSO_4_, 100 mM 2-N-morpholino ethanesulfonic acid [pH 5.7], 2 mM acetosyringone), and incubated for 2 h at room temperature. The suspensions were then adjusted to OD_600_=0.5 and infiltrated into 4- to 6-week-old wt or H2B-RFP transgenic *N. benthamiana* plants.

**TYLCV constructs for *Agrobacterium*-mediated inoculation**

The wt TYLCV infectious clone was constructed as previously described (Zhao et al., 2018). For the construction of an infectious clone of TYLCV containing V2^C85S^, a full length TYLCV mutant, TYLCV-C85S (the cysteine residue on V2 at amino acid 85 was changed to serine), was synthesized (Invitrogen, China). The full viral genomic DNA of TYLCV-C85S was amplified using the primers listed in S1 Table and inserted into the pGEM-T Easy (Promega, USA) vector to produce pGEM-1A-C85S. After sequence confirmation, a 2183 nucleotide (nt) fragment was excised from pGEM-1A with *Bam*HI and *Sac*I, then subcloned into the *Bam*HI-*Sac*I sites of the binary vector pBinPLUS to produce pBinPLUS-0.8A. The full-length of TYLCV-C85S was digested from pGEMT-1A with *Bam*HI and inserted into pBinPLUS-0.8A at its unique *Bam*HI site to get pBinPLUS-1.8A, the infectious clone of TYLCV-C85S.

**Nuclear-Cytoplasmic Fractionation Assay**

Nuclear-cytoplasmic fractionation assays were performed as described previously (Wang et al., 2011) with minor modifications. Infiltrated leaves were harvested and mixed with 2 mL/g of lysis buffer (20 mM Tris-HCl, pH 7.5, 20 mM KCl, 2 mM EDTA, 2.5 mM MgCl_2_, 25% glycerol, 250 mM Sucrose, 5 mM DTT and 10 mM protease inhibitor). The homogenate was filtered through a double layer of Miracloth. The supernatant, consisting of the cytoplasmic fraction, was centrifuged at 10,000 rpm for 10 min at 4°C and collected. The pellet was resuspended with 500 μL of NRB2 (20 mM Tris-HCl, pH 7.5, 0.25 M Sucrose, 10 mM MgCl_2_, 0.5% Triton X-100, 5 mM beta-mercaptoethanol and 10 mM protease inhibitor) and overlaid on top of 500 μL NRB3 (20 mM Tris-HCl, pH 7.5, 1.7 M Sucrose, 10 mM MgCl_2_, 0.5% Triton X-100, 5 mM b-mercaptoethanol and 10 mM protease inhibitor). These were centrifuged at 12,000 rpm for 40 min at 4°C. The final nuclear pellet was resuspended in 400 μL lysis buffer.

The cytoplasmic or nuclear fraction was mixed with 2×SDS loading buffer and boiled for 5 minutes. The samples were loaded onto a 12% (vol/vol) SDS/PAGE gel, proteins were transferred to a PVDF membranes, and target proteins were detected using a polyclonal anti-GFP antibody (GenScript, USA), a monoclonal anti-FLAG (Sigma, USA), a polyclonal anti-RFP antibody (GenScript, USA), or a polyclonal anti-PEPC antibody (Agrisera, Swedish), followed by anti-rabbit-IgG or anti-mouse-IgG secondary antibody conjugated to horseradish peroxidase (HRP) (Thermo Scientific, USA). The membranes were incubated in Supersignal West Femto substrate (Thermo Scientific) and the protein signals were detected using an Azure C400 ChemiDoc imager. The intensity of signals was quantified by using ImageQuant TL (GE healthcare). The signal was automatically identified, and the pixels values were measured, and compared with the background to obtain the signal intensity. The sum of intensity readings of the cytoplasm plus the nucleus was set as 100%. The values given are mean ± s.d. for repeats.

As quality controls for the fractionation assays, PEPC protein and H2B-RFP were used as a cytoplasmic and a nuclear marker, respectively.

**PTGS suppression analysis**

*Agrobacterium* harboring a functional GFP construct (pZAP-GFP) was infiltrated into GFP transgenic *N. benthamiana* plants at the 4-leaf stage. At 5 dpi, GFP-silenced plants were agroinfiltrated with constructs expressing TYLCV V2, V2^C85A^, V2^C85S^, or TBSV p19. For each construct, individual leaves were infiltrated with 500 μl of activated *Agrobacterium* culture (OD_600_ = 0.5). Photographs under UV illumination were taken 3 days after the second infiltration. Five leaves of GFP-silenced *N. benthamiana* plants were inoculated for each construct.

**Supplementary Table**

**S1 Table Primers used in this study**

|  | Designation | Sequence(5’ to 3’) | Assay |
| --- | --- | --- | --- |
| 1 | V1-bF | CGGGATCCATGTCGAAGCGACCAGGCGA | V1-YFP,  nYFP-V1 |
| 2 | V1-bR | CGGGATCCATTTGATATTGAATCATAGAAATAG | V1-YFP,  nYFP-V1,  FLAG-V1 |
| 3 | FLAG-V1-F | CGGGATCCATGGATTACAAGGATGATGATGATAAGTCGAAGCGACCAGGCGA | FLAG-V1 |
| 4 | V2-bgF | GAAGATCTATGTGGGATCCACTTCTAAAT | V2-YFP, cYFP-V2, V2^C85A^-YFP |
| 5 | V2-bgR | GAAGATCTGGGCTTCGATACATTCTGTAT | V2-YFP,  cYFP-V2, V2^C85A^-YFP, FLAG-V2,  FLAG-V2^C85A^ |
| 6 | FLAG-V2-F | GAAGATCTATGGATTACAAGGATGATGATGATAAGTGGGATCCACTTCTAAATG | FLAG-V2,  FLAG-V2^C85A^ |
| 7 | TY-V2-F | CGCCATATGATGTGGGATCCACTTCTAAAT | AD-V2,  AD-V2^C85A^,  AD-V2^C85S^,  AD-V2^C84AC86A^ |
| 8 | TY-V2-R | CGGAATTCTCAGGGCTTCGATACATTCT | AD-V2,  AD-V2^C85A^,  AD-V2^C85S^,  AD-V2^C84AC86A^ |
| 9 | TY-1A-KF | GGGGTACCACTTCTAAATGAATTTCCTGAATCTG | TYLCV-C85S |
| 10 | TY-1A-BR | CGGGATCCCACATAGTGCAAGACAAACT | TYLCV-C85S |
| 11 | SlSGS3-F | CGCCATATGAGTTTCAGCAAATGGGGTGGG | BD-SlSGS3 |
| 12 | SlSGS3-R | CGGGATCTCACATGGTGCCACTGCTATTGAC | BD-SlSGS3 |

**Supplementary Figure**

**S1 Fig** The formation of V2-YFP large aggregates in *N. benthamiana* cells is related to the concentrations of Agrobacteria used for infiltration. (a) Confocal microscopic images of V2-YFP-expressing cells from plants that have been infiltrated with Agrobacteria at OD_600_=0.5, 1.0 or 2.0. Arrows point to the aggregates in cells. Bars: 50 μm. (b) Measurements of large aggregates in cells expressing V2-YFP. Bars and numbers indicate the average numbers of aggregates as measured for an area of 4 mm^2^. In each treatment, large aggregates in 10 independent areas of 4 mm^2^ were counted.

**S2 Fig** Suppression of gene silencing by V2 protein in *N. benthamiana* line 16c. (a) GFP expression was silenced by infiltrating *Agrobaterium* harbouring a GFP expression construct. Photographs were taken under white light or UV light at 5 dpai. (b) Leaves of GFP-silenced plants were infiltrated with Agrobactria expressing V2, V2^C85A^, V2^C85S^, or p19 of TBSV. Photographs were taken under white light or UV light at 3 dpai. Five leaves were inoculated for each construct. (c) Quantitative RT-qPCR showing relative levels of GFP transcripts. The bars represent means ± SD. Data were obtained from three independent experiments.

**S3 Fig** Features of the V2^C85S^ mutant. (a) Co-IP assay showing that V2^C85S^ does not interact with the V1 protein. The Co-IP assay was performed as in Fig 2a. (b) The interaction between SlSGS3 and wt V2 or the V2^C85S^ mutant was examined in the yeast two-hybrid assay. The Y2H assay was performed as in Fig 4c. (c) The subcellular localization of the V1 protein that was co-expressed with FLAG-V2^C85S^ in *N. benthamiana* cells. DAPI stains DNA in the nucleus. Bars: 20 μm. The accumulated V1-YFP and FLAG-V2^C85S^ were detected by western blot analysis.
