# Supplementary figures and images for: Tomato Yellow Leaf Curl Virus V2 Protein Plays a Critical Role in the Nuclear Export of V1 Protein and Viral Systemic Infection
Source: Front Microbiol. 2020 Jun 10;11:1243. doi: 10.3389/fmicb.2020.01243 (PMC7297916; doi:10.3389/fmicb.2020.01243)

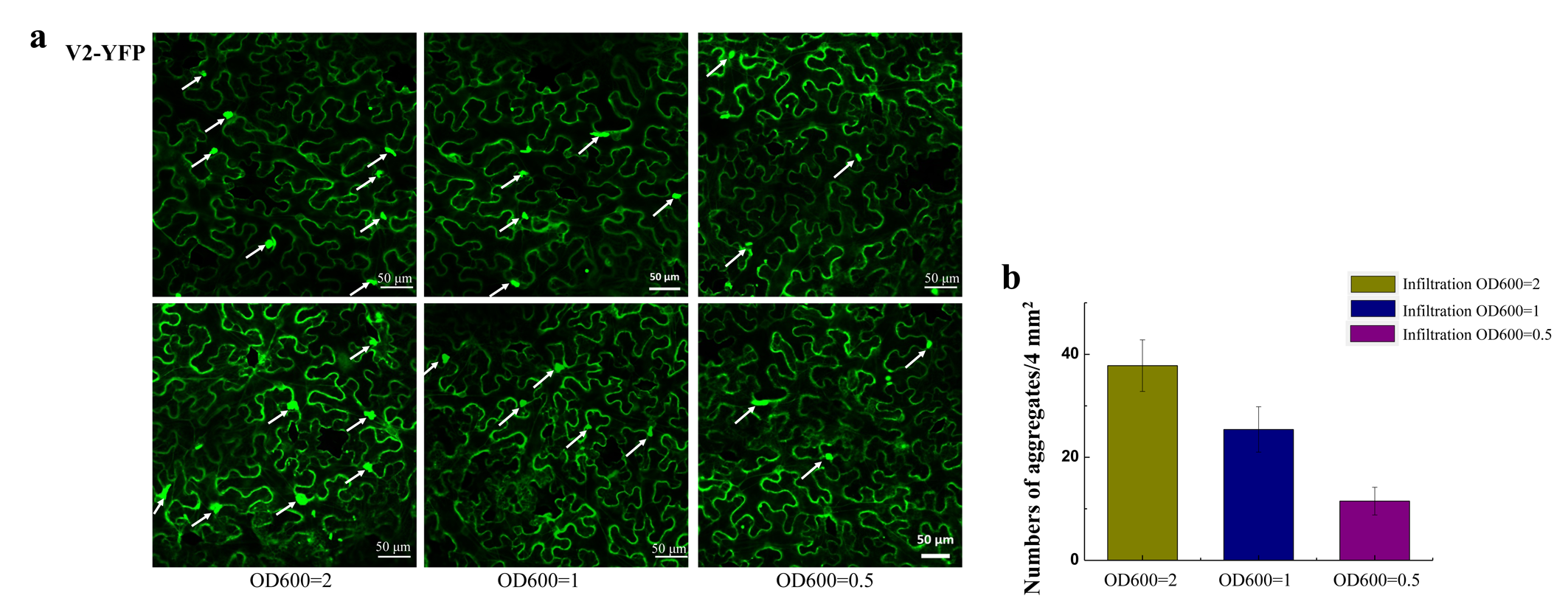

Supplement: Supplementary file 2 [file Image_1.TIF]

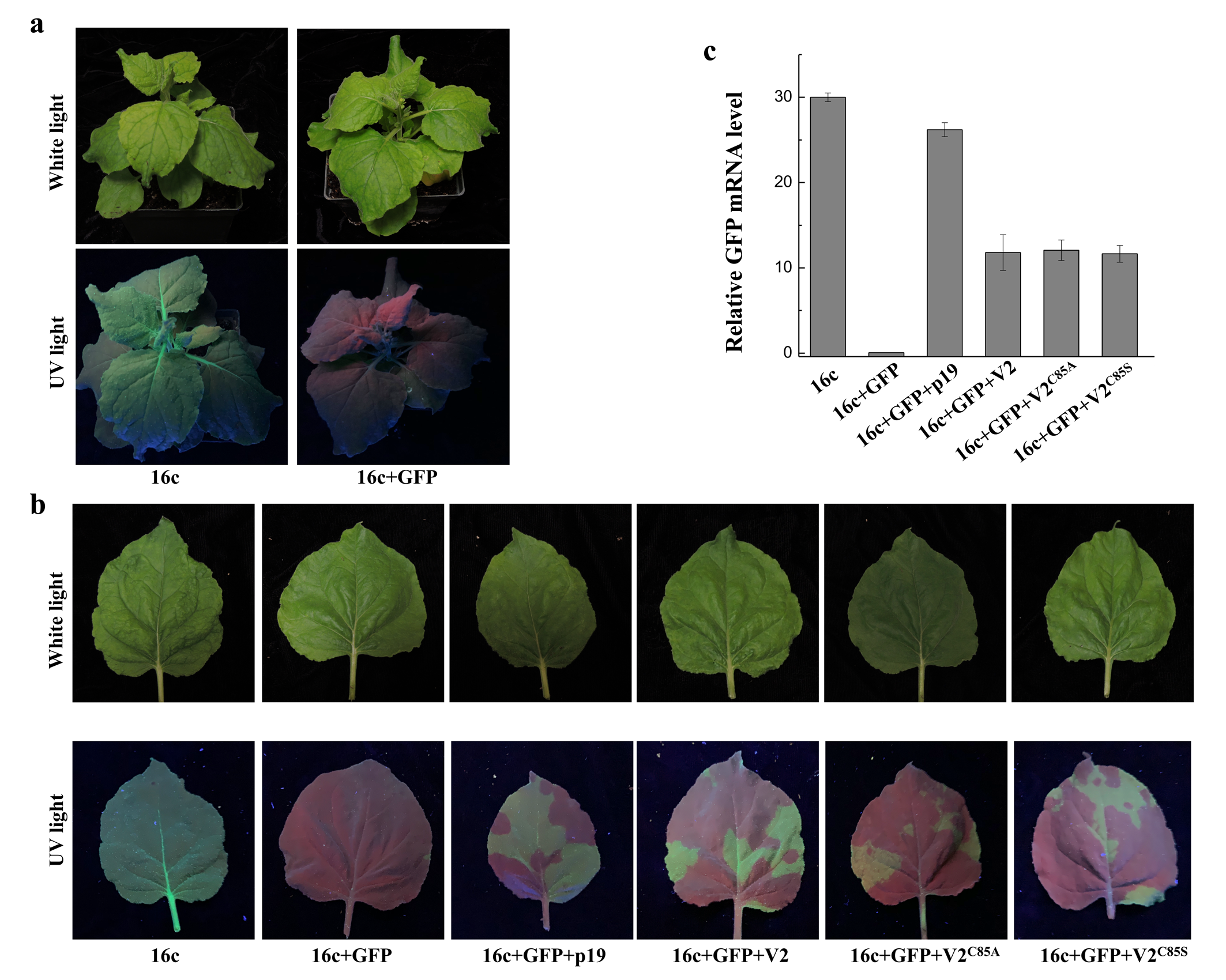

Supplement: Supplementary file 3 [file Image_2.TIF]

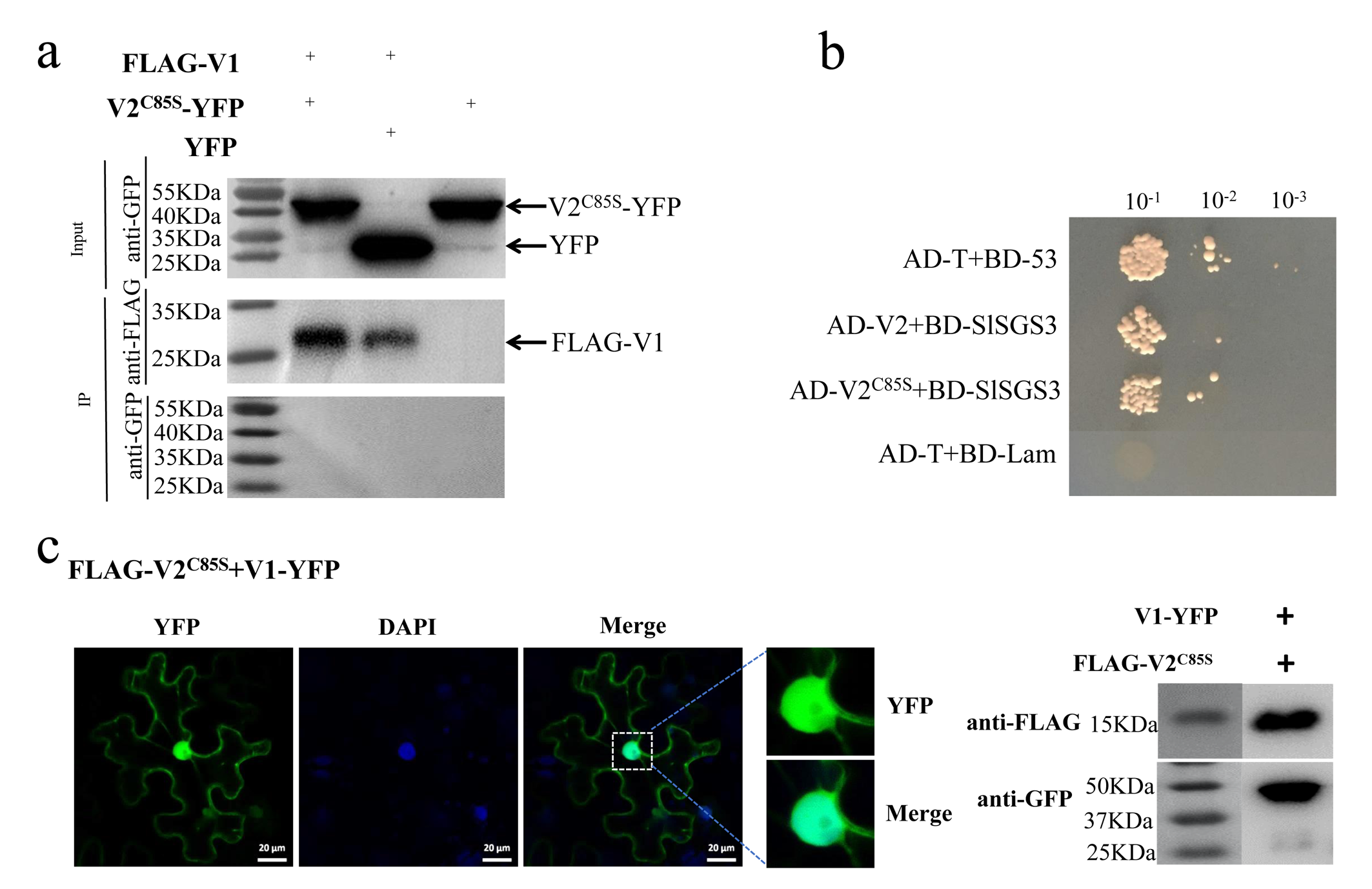

Supplement: Supplementary file 4 [file Image_3.TIF]
